# No Fans–No Pressure: Referees in Professional Football During the COVID-19 Pandemic

**DOI:** 10.3389/fspor.2021.720488

**Published:** 2021-08-19

**Authors:** Michael Christian Leitner, Fabio Richlan

**Affiliations:** ^1^Centre for Cognitive Neuroscience, University of Salzburg, Salzburg, Austria; ^2^Department of Psychology, University of Salzburg, Salzburg, Austria

**Keywords:** social pressure, decision making, referees, no fans, ghost games, football, home advantage, COVID-19

## Abstract

Due to the COVID-19 pandemic, European elite football (a.k.a. soccer) leagues played the remaining season 2019/20 without or strongly limited attendance of supporters (i.e., “ghost games”). From a sport psychological perspective this situation poses a unique opportunity to investigate the crowd's influence on referee decisions and the associated effect of “home advantage.” A total of 1286 matches–played in the top leagues of Spain, England, Germany, Italy, Russia, Turkey, Austria and the Czech Republic–were analyzed for results, fouls, bookings and reasons for bookings and contrasted between respective matchdays of season 2018/19 (regular attendance) and season 2019/20 (ghost games). Following recent methodological developments in the research on the home advantage effect, four different statistical analyses–including Pollard's traditional method–were used for the assessment of the home advantage effect. There are two main findings. First, home teams were booked significantly more often with yellow cards for committing fouls in ghost games. Most importantly, this effect was *independent* of the course of the games. In contrast, bookings for other reasons (criticism and unfair sportsmanship) changed similarly for *both* home and away teams in ghost games. Second, the overall home performance and home advantage effect in the respective elite leagues–identified in the respective matches of the regular 2018/19 season–vanished in the ghost games of the 2019/20 season. We conclude that the lack of supporters in top European football during the COVID-19 pandemic led to decreased social pressure from the ranks on referees, which also had a potential impact on the home advantage. Referees assessed the play of home teams more objectively, leading to increased yellow cards awarded for fouls committed by the home teams. Since there were no significant changes in referee decisions against the away teams, we argue that our observations reflect a reduction of unconscious favoritism of referees for the home teams.

## Introduction

Due to the COVID-19 pandemic, European top football (a.k.a. soccer) leagues paused competition around the middle of March 2020. The first league to restart was the “German Bundesliga” on May 16. Ultimately, 11 of 15 European's top leagues resumed competition and finished the season (Spain, England, Germany, Italy, Portugal, Russia, Ukraine, Turkey, Austria and Czech Republic). France, Belgium, Netherlands and Scotland suspended their leagues and aborted (“ORF Sport (2020). Frühlingserwachen in Europas Ligen,” 2020; “SPIEGEL Sport (2020). So planen Europas Fussballligen den Neustart,” 2020). The fundamental requirement for restarting competition was to play matches without the attendance of supporters; so-called “ghost games.” Only the Russian league chose to let a strongly limited number of supporters−10% of each stadium's capacity–attend matches (MDZ, 2020). From a psychological perspective, the circumstance of missing supporters on this unprecedented large scale in Europe's elite football leagues poses a unique situation in the history of professional football to investigate the crowds' influence on referees' decisions.

Allen and Jones (2014) state that “*a large body of research has confirmed that athletes and teams perform considerably better when they compete at home compared with away from home.”* (p. 48). Courneya and Carron (1992) define the home advantage as “*the term used to describe the consistent finding that home teams in sport competitions win over 50% of the games played under a balanced home and away schedule.”* (p. 13). Accordingly, Jamieson (2010) found in a meta study across sport disciplines that home teams win significantly more often than away teams. In this meta study, the strongest home advantage effects were found in Football (67.4%), followed by Rugby (63.7%), Basketball (62.9%), Tennis (61.5%), Boxing (60.8%), and Hockey (59.5%).

In the scientific literature, there is strong support for the hypothesis that the home advantage can be largely explained by social pressure on the referees, emanating from the present audience, attributed mainly to the noise the crowd produces in favor of the home team during games. Questions such as whether referees tend to favor the home team or disadvantage the away team are still subject of ongoing discussions (e.g., Pollard, 2008; Sors et al., 2020; Leitner et al., 2021; Wunderlich et al., 2021). From psychological and evolutionary perspectives, an explanation for the home advantage effect, based on social pressure and conformity are logical and comprehensible. Social psychological studies indicate that human beings tend to adapt to the opinion of the majority because individuals (unconsciously) believe that a group's interpretation of an ambiguous situation is more accurate than their own, ultimately leading to conform behavior (Cialdini and Goldstein, 2004). Accordingly, studies found that crowd size (Nevill et al., 1996), crowd density (Agnew and Carron, 1994; Goumas, 2012) and stadium properties (distance to the field) (Unkelbach and Memmert, 2010) positively correlate with the magnitude of the home advantage and corresponding the influence on referee decisions. In an experimental study, investigating the effects of crowd noise on refereeing decisions in football, it was found that referees–viewing the challenges with the crowds' noise–were more uncertain in their decision making and awarded significantly fewer fouls (15.5%) against the home team, compared with those referees evaluating the same game in silence (Nevill et al., 2002).

Other studies showed that the crowd size has a direct effect on the number of first yellow cards awarded to the away team in Cup final games (Downward and Jones, 2007), referees tend to issue red cards and award penalties significantly more often against the away team (Pollard and Armatas, 2017) and demonstrated the positive influence of training on referees' objectivity and decisions-making to overcome the effect of social pressure from the stands (Nevill et al., 2013). Before the COVID-19 pandemic forced the football world to play matches behind closed doors there was a study conducted on ghost games and the effects on refereeing. Specifically, a study from football games–that were played without attendance following safety requirements after hooligan incidents in Sicily (Italy)–showed that, under normal circumstances, home teams are favored by officials' decisions during matches (Pettersson-Lidbom and Priks, 2010). The authors state that “*the home team is punished less harshly than the away team across all outcomes in games with spectators [while] punished more harshly than the away team across all outcomes in games without spectators.”* (p.213). Further, regression analysis shows that the estimated bias effect is statistically significant for all outcomes regarding number of fouls, number of yellow cards and number of red cards. The authors conclude that their data “*strongly suggests that it is the referee that changes his behavior in games without spectators rather than the players.”* (p.214). There are, however, other studies suggesting that not only the referees but also the players can be emotionally influenced by the crowd, e.g., home players feeling motivated and away players feeling pressured (e.g., Almeida et al., 2011; George, 2015; Leite and Pollard, 2020).

By now, there are numerous studies from various fields regarding the effects of the COVID-19 pandemic on football, some of them concentrating on the home advantage effect. Overall, most of these studies indicate that the home advantage tends to decline when games are played behind closed doors (Bryson et al., 2020; Dilger and Vischer, 2020; Fischer and Haucap, 2020; Follert et al., 2020; McCarrick et al., 2020; Sors et al., 2020; Hill and Van Yperen, 2021; Konaka, 2021; Leitner et al., 2021; Sánchez and Lavín, 2021; Santana et al., 2021; Scoppa, 2021; Wunderlich et al., 2021). In this context, Bryson et al. (2020) state e.g., that “*without a crowd, fewer cards were awarded to the away teams, reducing home advantage [and that] these results have implications for the influence of social pressure and crowds on the neutrality of decisions.”* Other findings indicate that ghost games might also have a direct effect on the (non-verbal) behavior of professional football players, staff and officials on pitch during games (Leitner and Richlan, 2021). The authors found 19.5% fewer emotional situations in ghost games than in “regular games” (with fans present), meaning that players, staff and officials got less involved in behavior like “words fights” and “discussions” with each other.

Other (and also fewer) studies conclude that the home advantage effect did not change considerably during ghost games (Benz and Lopez, 2020; Almeida and Leite, 2021; Matos et al., 2021). Almeida and Leite (2021) emphasize that the effect of home advantage during the COVID-19 pandemic depends on the analysis level (individual league or across leagues) and that “*[…] the role of crowd support seems to vary depending on the context characteristics in which football is played*.” (p. 693).

Based on these previous and current studies investigating the absence of crowds in professional sports events, we hypothesized that the COVID-19 related ghost games in European elite football have significant effects on the officiating of referees. The present study examines and discusses these effects from a sport psychological perspective, while–as the first study of its kind–considering the course of the games for data analysis (i.e., the rewarding of yellow cards in relation to the current score of the game). Thus, the study provides novel insights regarding the crucial question of whether referees normally tend to advantage home teams or disadvantage away teams. We expected ghost games to have a substantial effect on fouls committed, number of cards awarded and an impact on the reasons for booking (criticism, unfair sportsmanship, foul play). In addition, we analyzed these factors in relation to the course of the games because we hypothesized to find–due to poorer performance of the home teams–peaks in the awarding of yellow cards for fouls depending on the current score (cf. Lago, 2009).

From a sport psychological perspective, the analysis of these performance and behavior parameters in the context of the dynamics of the game (i.e., current score) is of utmost importance. They not only reflect a potential bias in refereeing but are assumed to be based on underlying critical psychological states of players and officials (Courneya and Carron, 1992; Carron et al., 2005). Differences between regular games and COVID-19 related ghost games, therefore, are thought to particularly result from (less) perceived social pressure from the stands. In sum, the results of our study have substantial relevance for the research on social psychological processes built on conformity in sports. Possible practical applications are related to the development of training programs and interventions for referees to decrease the decision bias created by the home crowd.

## Methods

### Data Source and Variables

Based on the overall point value of UEFA's country coefficient list (UEFA, 2020a) we chose Europe's Top 15 leagues and excluded countries, in which leagues were suspended after the COVID-19 outbreak. The rationale for concentrating on these top leagues is that nations beyond rank 15 play a minor role in international football competition due to limited regular starters and a higher number of qualification rounds (up to five rounds with first and second leg). This plays a significant role when it comes to qualification for “UEFA Europa League” and especially for the top tier “UEFA Champions League” (UEFA, 2020b). Additionally, we selected only leagues that were statistically documented in detail on “transfermarkt.de,” which is an open and reliable statistics platform and regularly used as a source and forum for football related scientific studies (e.g., Franck and Nüesch, 2012). We analyzed the leagues' matches played after the respective restarts with no or significantly limited attendance (season 19/20) and compared the acquired data to the respective rounds (i.e., matchdays) of season 18/19, which were played with regular attendance. For an overview of the included and excluded league (see Table 1).

**Table 1:** Overview of the included and excluded leagues.

**League (County)**	**Included**	**Excluded (and reason for exclusion)**
La Liga (Spain)	x	
Premier League (England)	x	
1. Bundesliga (Germany)	x	
Serie A (Italy)	x	
Premier Liga (Russia)	x	
Süper Lig (Turkey)	x	
Tipico Bundesliga (Austria)	x	
Fortuna Liga (Czech Republic)	x	
Liga NOS (Portugal)		x (missing data in match statistics)
Premier Liga (Ukraine)		x (missing data in match statistics)
Superligaen (Denmark)		x (missing data in match statistics)
Ligue 1 (France)		x (aborted)
Jupiler Pro League (Belgium)		x (aborted)
Eredivisie (Netherlands)		x (aborted)
Scottish Premiership (Scotland)		x (aborted)

“La Liga” (Spain), “Premier League” (England), “1. Bundesliga” (Germany), “Serie A” (Italy), “Premier Liga” (Russia), “Süper Lig” (Turkey), “tipico Bundesliga” (Austria), and “Fortuna Liga” (Czech Republic) were included into the data sample. “Liga NOS” (Portugal), “Premier Liga” (Ukraine), and “Superligaen” (Denmark) were excluded from the study due to missing data in match statistics documentation regarding specific yellow card bookings. “Ligue 1” (France), “Jupiler Pro League” (Belgium), “Eredivisie” (Netherlands), and the “Scottish Premiership” (Scotland) aborted their seasons.

This led to a total sample size of 1286 matches, with 645 matches played in season 18/19 (regular attendance) and 641 matches played in season 19/20 (no or strongly limited attendance). The reason for fewer ghost games played in season 19/20 than regular games in season 18/19 can be found in the Russian “Premier Liga,” where four games were canceled due to COVID-19 cases (FK Orenburg vs. FK Krasnodar [Round 24], FK Orenburg vs. Ural Ekaterinburg [Round 25], FK Tambov vs. FK Sochi [Round 29] and Krylya Sovetov Samara vs. FK Sochi [Round 30]). Importantly, these four games were officially classified as uncontested by one of the teams (i.e., leading to a final score of 3:0) and therefore excluded from our analysis. As mentioned above, Russia was the only country to let an extremely limited number of supporters (10% of stadium capacity) attend football matches during the COVID-19 pandemic. Due to the significant discrepancy in crowd numbers between the two seasons we decided to include the Russian league into our analysis.

The following information was collected for each team and match played in the respective leagues: Result (win, loss, draw), points earned (0, 1, 3), goals scored, fouls committed, number of yellow, yellow-red and red cards awarded, reason for booking (criticism, unfair sportsmanship, foul play) and number of spectators.

### Statistical Analysis

Data was statistically analyzed on round-level, leading to an overall number of 73 rounds per season (*N* = 146). Due to ordinal scale of the data, non-parametric methods, two-sided Mann-Whitney-*U*-tests for independent data and Wilcoxon signed-rank test for dependent data were used to investigate potential differences in match statistics. Following recent methodological developments in the research on the home advantage effect, four different methods were used for the assessment of the home advantage effect: “Pollard's traditional method” (PTM), “Pollard's rescaled method” (PRM), “Stefani's method” (SM), and the “New method” (NM). These methods are excellently described in detail by Matos et al. (2020).

Home advantage (HA) is calculated as follows for the respective methodologies: HA(PTM) = [points won at home/(points won at home+points won away)]^*^100%; HA(PRM) = [(points won at home-points won away)/(points won at home+points won away)]^*^100%; HA(SM) = [(home wins-home losses)/total number of games]^*^100%; HA(NM) = [(points won at home-points won away)/points won away]^*^100%. Results from these home advantage calculations were tested with *t*-tests within season against each method's respective cut-off value representing “no home advantage” (PTM = 50%; PRM = 0%; SM = 0%; NM = 0%) and between seasons, respectively. Exact *p*-values are reported and statistical significance was defined by a *p*-value < 0.05. Effect sizes are reported in Pearson's *r*, calculated as follows: $r=\frac{z}{\sqrt{N}}$.

## Results

### Home Performance

**Figure 1:**
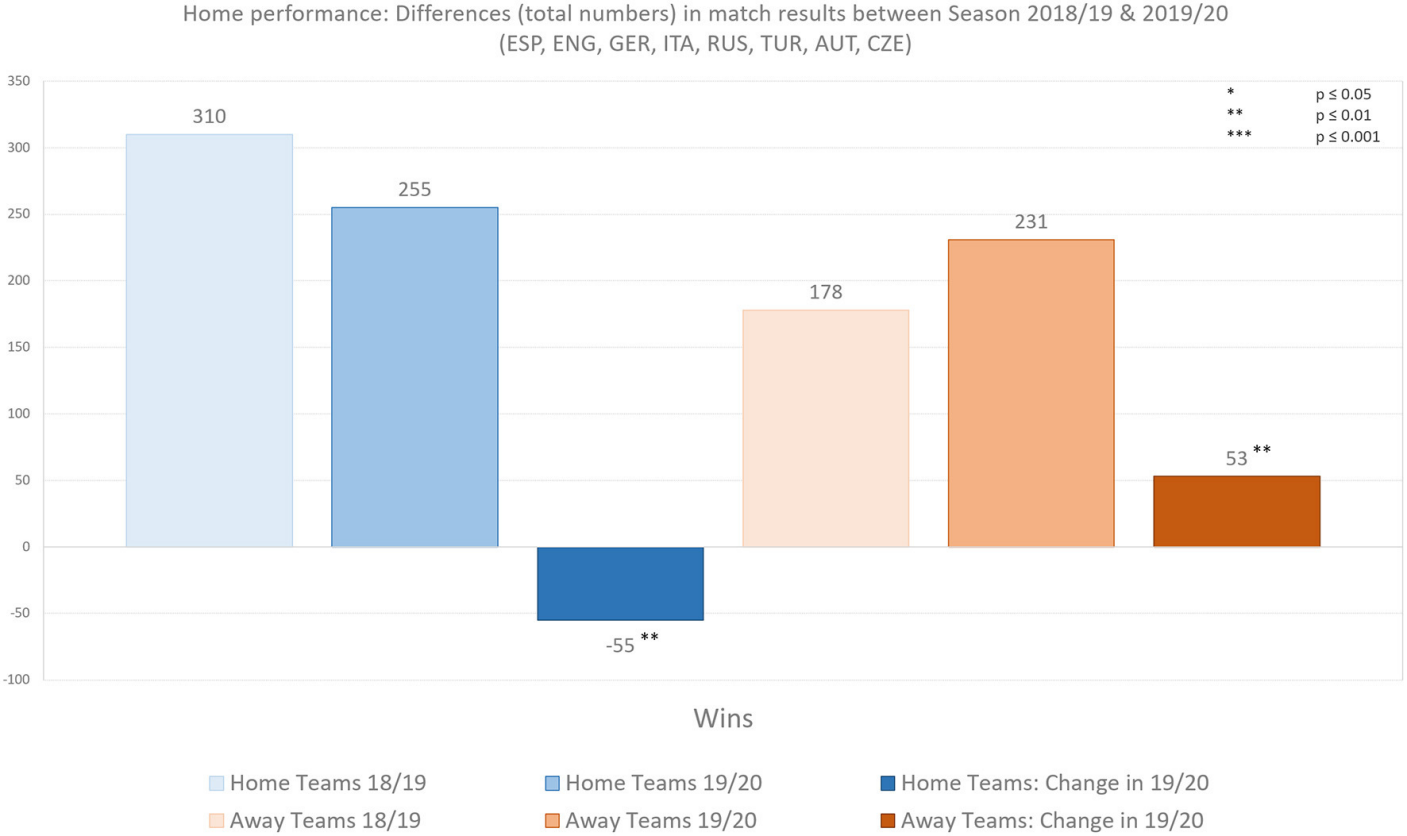
Absolute differences in home performance. Illustration shows the results of home and away teams in the highest football leagues of Spain, England, Germany, Italy, Russia, Turkey, Austria, and Czech Republic.

Figure 1 illustrates the total numbers and difference of match results statistics of the respective rounds analyzed between season 2018/19 and 2019/20. Home teams lost 55 (−17.7%) more games, away teams won 53 (+29.8%) more games, leading to 2 (−1.3%) fewer draws between regular games of 18/19 and ghost games of 19/20. We observed significantly fewer home wins (*U* = 1991, *p* = 0.007, *r* = 0.222) and more away wins (*U* = 1913, *p* = 0.003, *r* = 0.249) between regular matches of season 18/19 and ghost games of season 19/20.

**Figure 2:**
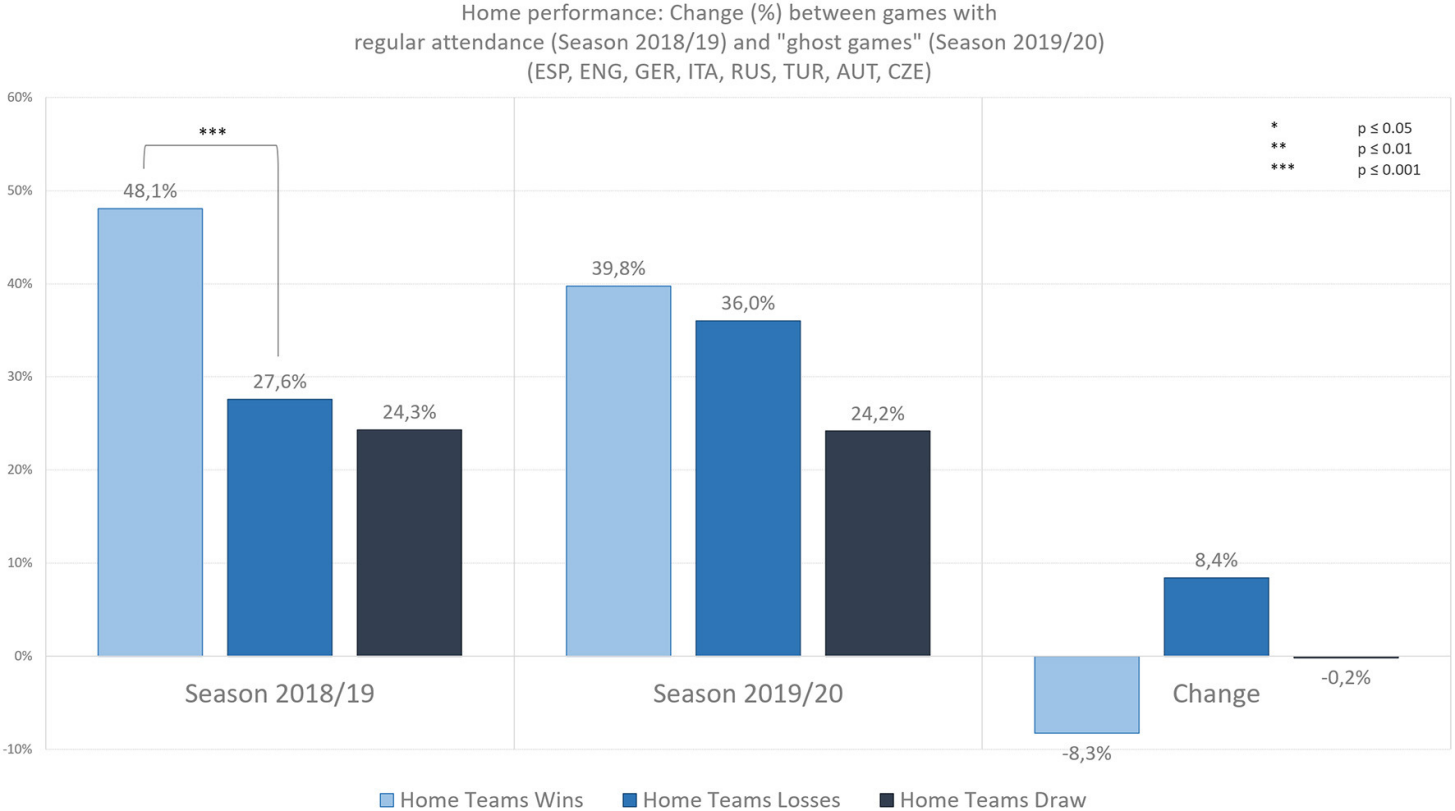
Relative changes of home performance in games with regular attendance (season 2018/19) and ghost games (season 2019/20) in the highest football leagues of Spain, England, Germany, Italy, Russia, Turkey, Austria, and Czech Republic.

Figure 2 illustrates the relative change in team performance when playing at home between seasons of 2018/19 (regular attendance) and 2019/20 (ghost games). Home teams won 48.1%, lost 27.6%, and drawed in 24.3% of all analyzed games with regular attendance in season 2018/19. This difference between the number of home wins compared to home losses is statistically significant. Therefore, with regular attendance, home teams won significantly more often (than they lost) in home games (*z* = −5.376, *p* = 0.000, *r* = 0.445). In contrast, in the ghost games of season 2019/20, home teams won 39.8%, lost 36.0% and drawed 24.2% of all analyzed matches. This difference between the number of home wins compared to home losses is not significant. Therefore, in ghost games, home teams did not win significantly more often (than they lost) in home games (*z* = −1.264, *p* = 0.206, *r* = 0.105). It should be noted at this point that we decided against excluding draws as in other studies on home performance (e.g., Jamieson, 2010). If calculated without draws (i.e., taking into consideration only the games that resulted in a win or loss), the home performance increases to 63.5% wins in season 2018/19 (which is in line with the findings of the meta-analysis by Jamieson, 2010) and 52.5% wins in season 2019/20.

### Home Advantage

**Figure 3:**
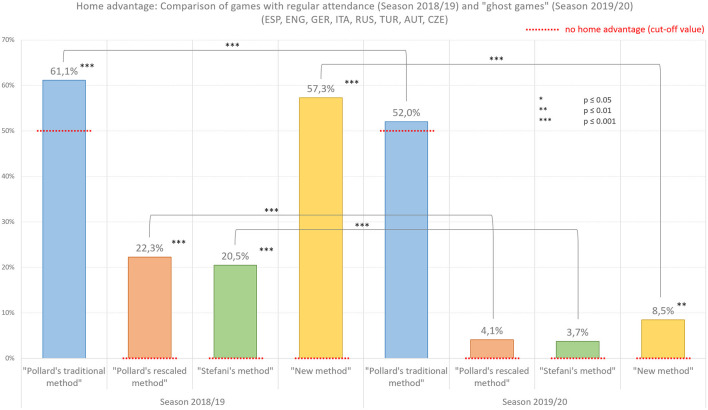
Difference in home advantage between matches with regular attendance in season 2018/19 and ghost games in season 2019/20, calculated with four different methods.

In order to represent the change in home advantage in the course of the ghost games of season 2019/20 as comprehensively and accurately as possible, we used four different calculation methods, as illustrated in Figure 3. Each of these methods is used in various sports science publications and have different advantages and disadvantages (see Matos et al., 2020 for a detailed description). “Pollard's traditional method” varies from 0% (no points won at home) to 100% (no points won away), 50% representing no home advantage. “Pollard's rescaled method” results in 0% when there is no home advantage, with a maximal value of 100% for home advantage and a maximal value of −100% for home disadvantage. “Stefani's method” varies from 0%–representing no home advantage–to 100% and “New method” ranges from −100% (home disadvantage) to *over* 100% (home advantage).

There is a statistically significant difference in all four methods when testing home advantage results of regular games in season 2018/19 against the method's respective “no home advantage cut-off values”: Pollard's traditional method: [*t*(72) = 6.350, 95% CI [0.074, 0.142], *p* = 0.000, *r* = 0.599]; Pollard's rescaled method: [*t*(72) = 6.297, 95% CI [0.147, 0.283], *p* = 0.000, *r* = 0.596]; Stefani's method: [*t*(72) = 6.200, 95% CI [0.134, 0.261], *p* = 0.000, *r* = 0.590]; New method: [*t*(72) = 6.142, 95% CI [0.661, 1.297], *p* = 0.000, *r* = 0.586]. When testing the home advantage results of the ghost games in season 2019/20 there are no significant differences in three of four methods from the method's respective “no home advantage cut-off values”: Pollard's traditional method: [*t*(72) = 0.740, 95% CI [−0.023, 0.0501], *p* = 0.462, *r* = 0.087]; Pollard's rescaled method: [*t*(72) = 0.725, 95% CI [−0.046, 0.099], *p* = 0.471, *r* = 0.085]; Stefani's method: [*t*(72) = 0.685, 95% CI [−0.044, 0.090], *p* = 0.496, *r* = 0.080]; New method: [*t*(72) = 3.083, 95% CI [0.103, 0.482], *p* = 0.003, *r* = 0.341].

When comparing the home advantage results of the regular games of season 2018/19 with the results of the ghost games of season 2019/20 there is a statistically significant difference in all four methods: Pollard's traditional method: [*t*(144) = 3.780, 95% CI [0.045, 0.143], *p* = 0.000, *r* = 0.300]; Pollard's rescaled method: [*t*(144) = 3.772, 95% CI [0.089, 0.286], *p* = 0.000, *r* = 0.300]; Stefani's method: [*t*(144) = 3.772, 95% CI [0.832, 0.266], *p* = 0.000, *r* = 0.300]; New method: [*t*(144) = 3.701, 95% CI [0.320, 1.053], *p* = 0.000, *r* = 0.295].

### Fouls and Yellow Cards

From a sport and social psychological perspective, the analysis of fouls and cards is even more interesting than the analysis of match results, because these parameters are more proximal to actual displayed (mis)behavior and, subsequently, to underlying critical psychological states. Aggregation of all committed fouls from all analyzed matches of the eight included leagues and comparison between the two seasons of 2018/19 (regular games) and 2019/20 (ghost games) shows that both home (+296 fouls/+3.7%) and away (+75 fouls/+0.9%) teams committed more fouls in ghost games (in absolute numbers). There is a significant increase in total fouls committed by home teams (*U* =2143, *p* = 0.041, *r* = 0.169) but no significant difference in total fouls committed by away teams (*U* = 2365, *p* = 0.240, *r* = 0.097) between regular matches of season 18/19 and ghost games of season 19/20. In addition, in ghost games, the total number of yellow cards awarded to the home teams increased (+148 yellow cards/+11.7%), whereas it decreased for the away teams (−77 yellow cards/−5.5%). There is no significant difference in the total yellow cards awarded to home teams (*U* = 2170, *p* = 0.053, *r* = 0.160) and the total yellow cards awarded to away teams (*U* = 2409.5, *p* = 0.317, *r* = 0.083) between regular matches of season 18/19 and ghost games of season 19/20.

**Figure 4:**
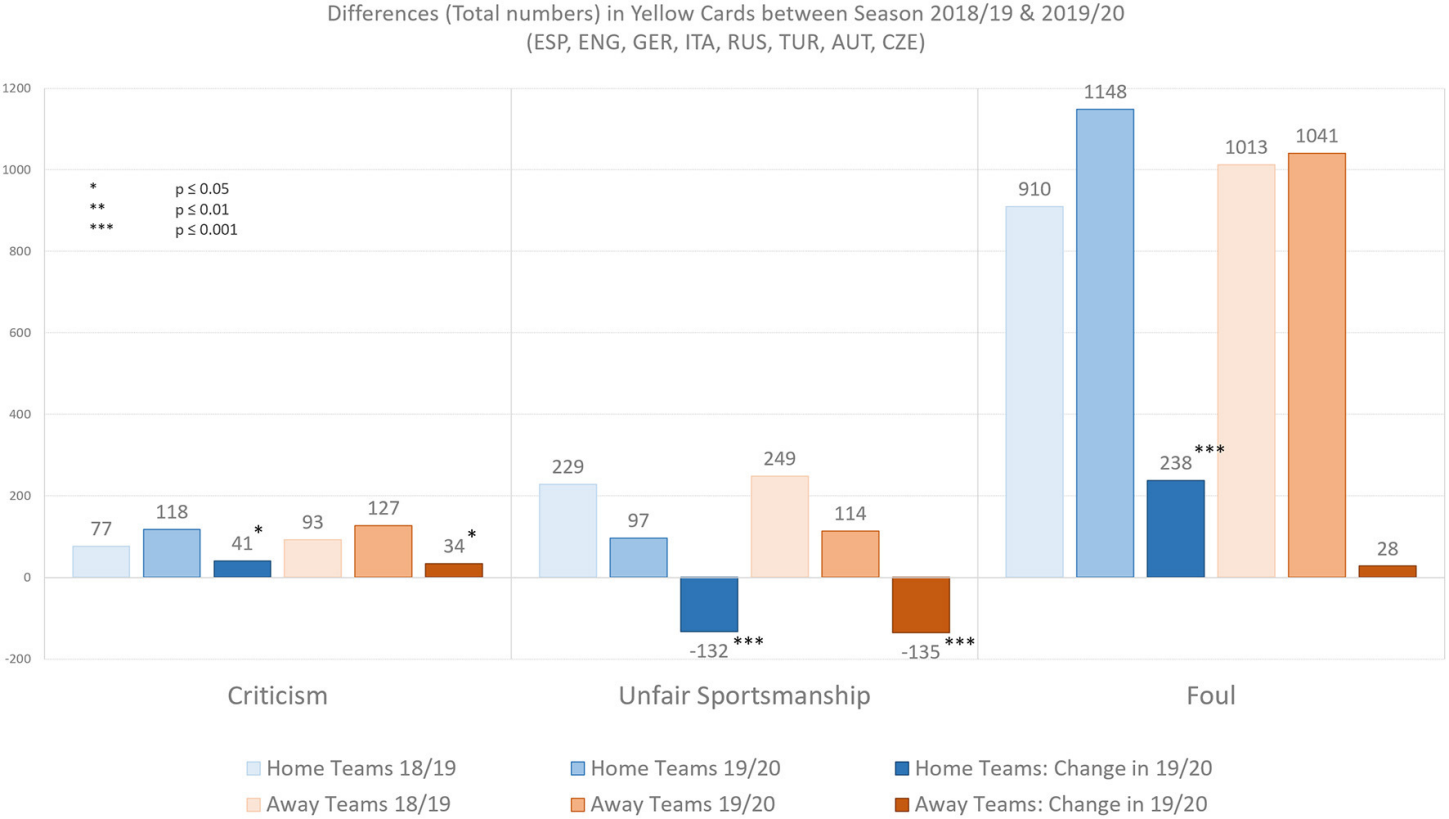
Absolute differences in Criticism, Unfair Sportsmanship, and Foul Play. Illustration shows yellow cards (criticism, unfair sportsmanship, and foul play) awarded to home and away teams in the top football leagues of Spain, England, Germany, Italy, Russia, Turkey, Austria, and Czech Republic. Bar plots are based on aggregated match results statistics of rounds played in the leagues as ghost games (no or strongly limited attendance) in season 2019/20 and respective rounds of regular matches (regular attendance) in season 2018/19 (*N* = 146).

Figure 4 illustrates the total numbers and difference of yellow cards awarded for criticism, unfair sportsmanship and foul play of the respective rounds analyzed between season 2018/19 and 2019/20. Comparing absolute and relative numbers of yellow cards awarded for criticism, unfair sportsmanship, and foul play for home and away teams between regular games of season 18/19 and ghost games of season 19/20 showed the following: yellow cards for criticism (home teams: +41/away teams: +34) increased by 53.2% for home teams and by 36.6% for away teams. Unfair sportsmanship (home teams: −132/away teams: −135) decreased by −57.6% for home teams and by −54.2% for away teams. Contrarily and most interestingly, yellow cards awarded for fouls increased strongly for home teams (+238) by 26.2% but only slightly (+28) for away teams (+2.8%).

Statistical analysis reveals that there were significantly more yellow cards awarded for criticism for the home teams (*U* = 2057.5, *p* = 0.013, *r* = 0.205) and for the away teams (*U* = 2173, *p* = 0.048, *r* = 0.164) between regular matches of season 18/19 and ghost games of season 19/20. Furthermore, there were significantly fewer yellow cards awarded for unfair sportsmanship for the home teams (*U* = 1297, *p* = 0.000, *r* = 0.451) and for the away teams (*U* = 1513.5, *p* = 0.000, *r* = 0.380) between regular matches of season 18/19 and ghost games of season 19/20. Finally, there was a significant difference in more yellow cards awarded for foul play for the home teams (*U* = 1712.5, *p* = 0.000, *r* = 0.309) but *no* significant difference in yellow cards awarded for foul play for the away teams (*U* = 2497.5, *p* = 0.513, *r* = 0.054) between regular matches of season 18/19 and ghost games of season 19/20.

**Figure 5:**
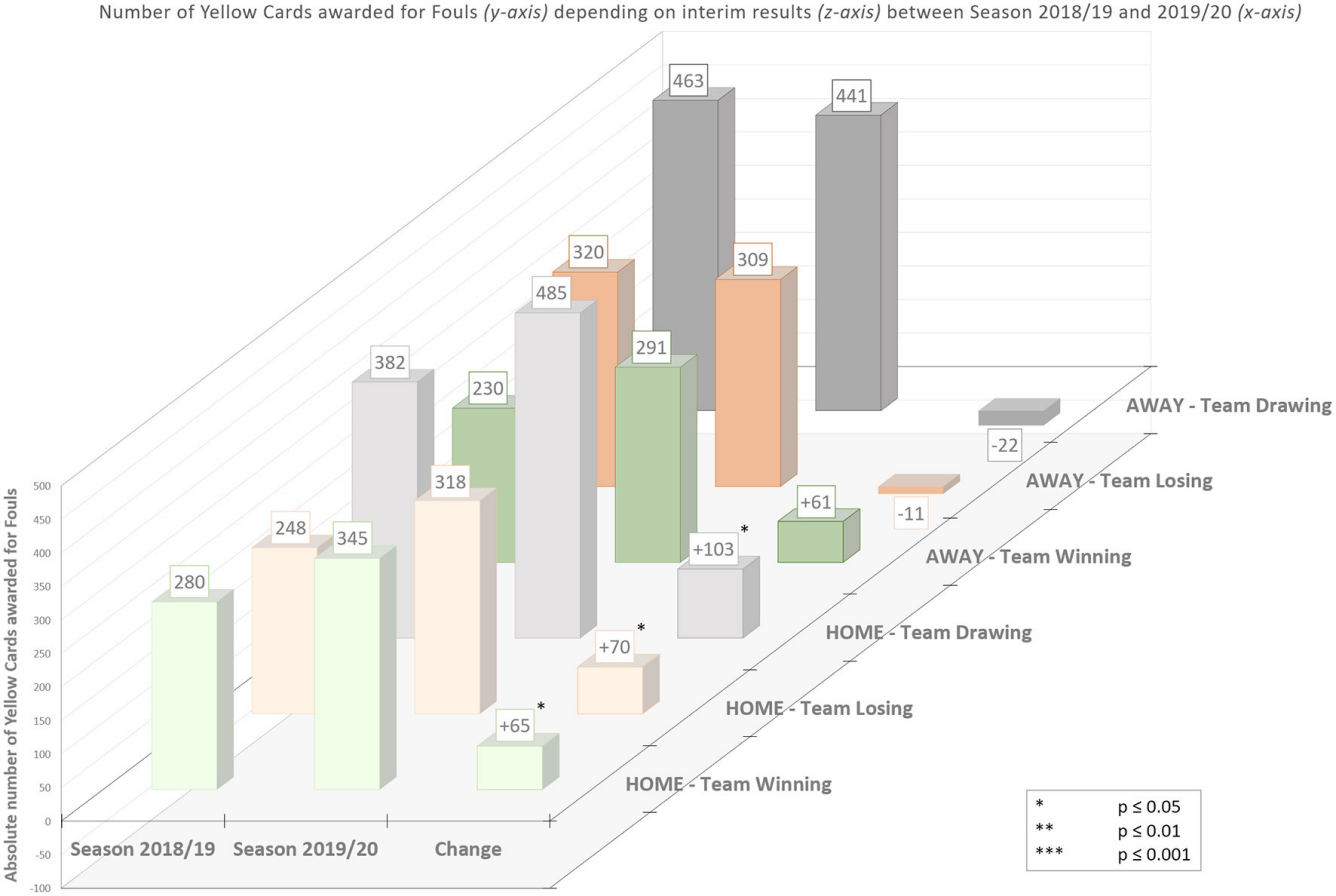
Absolute differences in yellow cards for foul play depending on the course of the game. Absolute differences of yellow cards of home and away teams while winning, losing, or drawing in respective games of season 2018/19 (regular attendance) and season 2019/20 (limited attendance).

Due to the findings of a significantly increased number of yellow cards awarded for fouls, we performed an in-depth analysis by collecting data on the relationship between course of play and the awarding of yellow cards. We found this effect (i.e., the higher number of yellow cards for fouls) to be independent of the current score, but dependent on the team (home or away). Figure 5 illustrates that, regarding yellow cards awarded for foul play, every condition of the current score (winning, losing, drawing) of the home team differs significantly in season 2019/20 from season 2018/19, while there is no significant effect for any condition of the away team.

Comparing the two seasons, we observed a significantly higher number of yellow cards awarded for fouls for the home teams while leading (*U* = 2060.5, *p* = 0.017, *r* = 0.197), but not for the away team while leading (*U* = 2195, *p* = 0.063, *r* = 0.154) in ghost games of season 19/20. Furthermore, there is a significant difference in more yellow cards awarded for fouls for the home teams while trailing (*U* =2100, *p* = 0.026, *r* = 0.184) but not for the away teams while trailing (*U* = 2623.5, *p* = 0.872, *r* = 0.013) in ghost games of season 19/20. Similarly, we observed significantly more yellow cards awarded for fouls in ghost games of season 19/20 for the home teams while the score was draw (*U* = 2034, *p* = 0.013, *r* = 0.205) but not for the away teams (*U* = 2564.5, *p* = 0.694, *r* = 0.033).

## Discussion

The main finding of our study is that home teams are awarded with significantly more yellow cards for committed fouls in ghost games than in regular games with supporters present. In contrast, the number of yellow cards for fouls awarded to away teams is unaffected by ghost games. Conversely, other rule infringements awarded with yellow cards increase (criticism) or decrease (unfair sportsmanship) similarly for both home and away teams.

Our findings on significantly decreased yellow cards awarded for unfair sportsmanship are in line with results from recent research, where it was found that ghost games have an overall calming effect on players, staff and officials, with an increase in fair-play behavior (Leitner and Richlan, 2021). Regarding the significant increase in yellow cards awarded for criticism, we argue that this represents another effect which was also found in the same study: referees tend to engage themselves significantly less during protest or words fight behavior with players. The increase in yellow cards for criticism reflects the referee's behavior to stop nascent discussions because they feel less obliged to justify their decisions to the players due to the lack of social pressure from the audience. We further analyzed the course of the games and documented at what point in the matches yellow cards were awarded for fouls. Our initial assumption was that–due to the average worse performance of the home teams in ghost games–the increased number of yellow cards was due to bad performance, i.e., trailing in score. Our analyses, however, revealed that this was not the case and that a significant increase in yellow cards awarded for fouls could be observed in *all* three interim score conditions (winning, losing, drawing).

The second finding of our study is that overall, football teams of Europe's elite leagues, lose significantly more of their home games and win significantly more of their away games, when there are no supporters in the stadium attending the matches. This finding is in line with other current studies (for an overview see Leitner et al., 2021). Corresponding evidence was also identified in our study in the games with supporters taking place in the 2018/19 season, with a quantified home performance of 63.5% home-win-rate or a plus of 0.61 points per game for home (1.69 points per game) relative to away (1.07 points per game) teams. Without supporters on matchday, home performance diminishes to a 52.5% home-win-rate or a plus of only 0.11 points per game for home (1.44 points per game) relative to away (1.32 points per game) teams. Interestingly the number of draws appears to be stable and unaffected by ghost games.

Further statistical analyses based on four different methods for assessing home advantage (Matos et al., 2020) indicate that the otherwise well-documented effect of home advantage in football is significantly reduced in ghost games. The two best known statistical approaches for calculating home advantage– “Pollard's traditional method” and “Pollard's rescaled method” – both show a highly significant change in home advantage in the course of ghost games. The results from “Stefani's method” also suggest that the absence of fans in the course of the 2019/20 season had a statistically significant impact on the game. Only “New method” does not generate statistical significance when compared to the respective cut-off value, but the inter-seasonal comparison (i.e., season 2018/19 vs. season 2019/20) is highly significant here as well. We therefore argue that the overall picture suggests a substantial change toward decreasing home advantage in the course of the ghost games of the 2019/20 season.

We argue that due to missing supporters, referees act–based on the theories and findings of e.g., Nevill et al. (2002, 2013), Pettersson-Lidbom and Priks (2010) and Unkelbach and Memmert (2010)–more objectively in their decision-making during ghost games, which leads to consequent booking of home teams after rule infringement. As concluded by Pettersson-Lidbom and Priks (2010) ghost games decrease the effect of social pressure from the stands and therefore lead to changes in refereeing and officials' behavior. Thus, our results suggest that referees–when spectators are present–normally tend to advantage the home team. This is because our analysis shows that during ghost games, only home teams are significantly more awarded with yellow cards for fouls and not away teams. Our findings and conclusions are consistent with the experimental study by Nevill et al. (2002), who found referees–watching a recorded football game–to significantly decide more accommodating for the home team when the home crowd's acoustic reactions were hearable in ambiguous and critical situations (such as fouls) than when TV audio was turned off. In contrast, decisions against the away team were not significantly different in both conditions (noise vs. no noise from the home crowd).

The scientific literature shows that in controversial and ambiguous situations human beings tend to (unconsciously) rely on the opinion of the majority–in this case the crowd supporting the home team. This subconscious preference for the home team probably represents a crucial part of the home advantage effect. Especially against the background of our study showing that the course of the game does not play a role in the awarding of yellow cards (see Figure 5), the results allow such a conclusion. In particular, the evaluation of tacklings is one of the most challenging decisions for referees in football. The rating of these situations must be fulfilled in a consistent manner and in the fractions of a second during the dynamic of the game. We argue that especially this narrow time window to make quick decisions in a game that has become faster and more intense in recent years is the crucial factor for the influence of the crowd on referees' decisions. Referees find it more and more difficult to correctly oversee and evaluate the increasingly fast-paced situations, as football in general has become progressively athletic and intense, also for tactical reasons. Robert Sedlacek, the head of referees in the Austrian Bundesliga recently stated that “*[Football] gets faster, harder and more intense. The line between foul and show is getting narrower and narrower*.” (DerStandard, 2021).

Intensive pressing schemes, which–instead of being played dynamically for rather short phases of the game in the past–are now part of the basic and ongoing tactical orientation of many clubs. These developments also have favored the introduction of the so-called video assistant referee (VAR). Because due to the increased general dynamics of the game, many decisions–which potentially have a massive influence on the outcome of games–can no longer be made satisfactorily reliably without technical aids. For an excellent overview of the effects of the introduction of the VAR on the game see Lago-Penas et al. (2019). The introduction of video evidence, however, does not play a role for the present study because the VAR may only intervene in the following four basic situations when the initial decision by the referee is evidently wrong: (1) Goal (2) Penalty (3) Red card (4) Player mix-up (in awarding a yellow, yellow-red or red card). However, the incorrect awarding of a yellow card for a (supposed) foul is no reason for the VAR to intervene.

### Practical Applications

We argue that our finding of referees tending to advantage home teams in matches with supporters leads to novel approaches for developing efficient methods and interventions in the training of referees. It is conceivable that multistage procedures in such a training, comprising of the following central training contents, could lead to success to better protect against the influence of social pressure from the crowd: (1) Presentation of the scientific findings regarding social pressure on the experience and perception of referees; (2) Raising awareness about the significant effect of social pressure on objective refereeing and the result of home team advancement; (3) Development of Virtual Reality Trainings that enable a deeper and direct experience of refereeing, accompanied with individual feedback and training regarding the correctness of the decisions made in controversial situations.

Although we assume that the COVID-19 pandemic has most likely a general psychological impact on the everyday experience of people worldwide–originating from uncertainty and fear–it is highly unlikely the sole reason for changed on-pitch-behavior of protagonists in professional football. Rather, it is more probable that situational factors during competition have a more substantial impact on the experience of referees. Because the most important matchday-related difference between regular matches in late stages of season 2018/19 and ghost games in late stages of season 2019/20 is the missing crowd and its external stimulation, we assume that our documented effects of decreased home advantage (in line with many other studies; for a review see Leitner t al., 2021) and increased yellow card booking for fouls for home teams mainly attribute to the missing supporters in the stadium.

### Limitations

We cannot exclude the possibility that ghost games have a substantial impact on the “aggression potential” of players. Theoretical models and studies exist on the assumption that home advantage reflects an evolutionary behavior of territorial defense. In this context, studies show that players of home teams have higher testosterone levels than players of visiting teams (e.g., Neave and Wolfson, 2003). It is reasonable to assume that the missing of a home crowds' support creates a void in this territorial defense mechanics that must be compensated by some sort, changing the on-pitch behavior of the home team players. This effect could result in increased (dysfunctional) aggressive behavior, which, in turn, results in more fierce tackles, fouls and ultimately in more bookings for fouls. This hypothesis, however, is contradictory to results from a recent study, which shows that about 20% fewer emotional situations can be observed between players, coaches, and referees due to the lack of an audience (Leitner and Richlan, 2021). Similarly, we cannot rule out the possibility that other factors have changed during ghost games that have contributed to a flattening of the home advantage. No further conclusions can be drawn from the data available to us. However, based on previous and current studies and experiments already conducted on this topic (see above for details), the empirical basis for the assumption that referees play a large role in the effect of home advantage is evident.

In contrast to other studies, we decided not to analyze the data on league level or include a vast number of different (worldwide) leagues. The reason is the deliberately focused scope of our study, concentrating on both economic and sporting top tier European leagues. As mentioned above, European top 15 leagues are the most important football leagues in the world, with regular attendance in major tournaments such as UEFA Champions League and UEFA Europa League. In our view it is not sensible to mix elite leagues and minor leagues (as second or even third leagues) when investigating complex sport psychological phenomena like home advantage and underlying mechanisms regarding the effects of (big) crowds on referee decisions. In our opinion, a more focused and controlled approach is more favorable from an empirical standpoint.

Additionally, we want to note that there are various possible approaches regarding the time frames for contrasting match data from different rounds of football played over different seasons in different countries. Because we believe that stretching the time horizon for analysis creates inaccuracies, we decided to compare the ghost games of season 2019/20 only with games from the same rounds of the previous season of 2018/19. For example, the comparison of two consecutive seasons decreases biases such as evolution of tactics, rules, league phases, play level of teams and leagues. We are fully aware that there are other research groups dealing with this matter differently, but we strongly believe that our approach stands on reasonable empirical grounds, particularly considering the remarkably fast development of elite football.

Using our approach, however, it is impossible to control for competitive balance and/or relative strengths of the teams and their opponents, because there were different pairings in the analyzed rounds of the two seasons. Actually, controlling for competitive balance is a difficult endeavor in the context of the fast-evolving sport of football. In particular, one may argue that the relative strength of teams changes dynamically across two consecutive seasons or even within single seasons. For example, in the Austrian Bundesliga we usually observe a marked difference in the performance of teams between the fall and the spring sub-parts within a season. Consequently, any comparison between two teams differs with respect to relative strength from a comparison of the same teams at a different point in time.

## Conclusions

Our results suggest that ghost games (without spectators) of professional elite football during the COVID-19 pandemic differ significantly from regular games (with spectators). Firstly, the analysis of yellow cards for fouls indicate that referees give less preferential treatment to the home team in ghost games than in regular games. Secondly, the absence of spectators significantly reduces the home advantage. We argue that a link between these two effects is obvious–also in light of existing scientific work from the past. We reason that due to the missing supporters in ghost games, referees perceived less social pressure from the home crowd, leading to dissolvement of the home advantage effect. Referees may act more objectively in their decision making regarding ambiguous situations such as the evaluation of tacklings, leading to a significant rise of yellow cards awarded for fouls only for home teams but *not* for away teams. This indicates that referees may give the home teams an (unconscious) advantage in games with regular attendance–contributing to the effect of home advantage–while *not* disadvantaging the away teams. Our findings go well in line with conclusions from previous research. Finally, our research results provide an empirical basis for the development of training methods to minimize the influence of the audience on referees' decision making in the future.

## Data Availability Statement

Publicly available datasets were analyzed in this study. This data can be found at: www.transfermarkt.de.

## Author Contributions

All authors listed have made a substantial, direct and intellectual contribution to the work, and approved it for publication.

## Conflict of Interest

The authors declare that the research was conducted in the absence of any commercial or financial relationships that could be construed as a potential conflict of interest.

## Publisher's Note

All claims expressed in this article are solely those of the authors and do not necessarily represent those of their affiliated organizations, or those of the publisher, the editors and the reviewers. Any product that may be evaluated in this article, or claim that may be made by its manufacturer, is not guaranteed or endorsed by the publisher.
